# Socioeconomic Disparities in Prevalence, Treatment, and Control of Hypertension in Middle-aged Koreans

**DOI:** 10.2188/jea.JE20110132

**Published:** 2012-09-05

**Authors:** Sun Hwa Cha, Hye Soon Park, Hong Jun Cho

**Affiliations:** Department of Family Medicine, Asan Medical Center, University of Ulsan College of Medicine, Seoul, Korea

**Keywords:** hypertension, prevalence, treatment, control of hypertension, socioeconomic disparities

## Abstract

**Background:**

We investigated socioeconomic inequalities in hypertension prevalence, treatment, and control among middle-aged Koreans.

**Methods:**

We analyzed data from 4275 adults aged between 40 and 64 years who participated in the Korean National Health and Nutrition Examination Survey, 2007 and 2008. Education, income, and occupational level were evaluated to assess the relationship of socioeconomic status with hypertension prevalence, treatment, and control.

**Results:**

There were significant differences in socioeconomic status among individuals with no hypertension, controlled hypertension, and uncontrolled hypertension in both sexes. In multiple logistic regression models, as compared with men who had more than 12 years of education, those with 7 to 12 years and less than 7 years of education had odds ratios (ORs) for untreated hypertension of 2.14 (95% CI: 1.18 to 3.90) and 2.98 (95% CI: 1.42 to 6.28), respectively (*P* for trend <0.05). As compared with women who had more than 12 years of education, those with 7 to 12 years and less than 7 years of education had ORs for hypertension prevalence of 1.75 (95% CI: 1.10 to 2.78) and 1.88 (95% CI: 1.12 to 3.16), respectively (*P* for trend <0.05). Women who worked as manual labors had an OR for uncontrolled hypertension of 1.50 (95% CI: 1.02 to 2.22) as compared with women in other jobs. There was no statistically significant association between income level and hypertension control.

**Conclusions:**

Socioeconomic status was independently associated with hypertension prevalence and care, which suggests a need for health policy efforts to reduce the socioeconomic disparity in hypertension management.

## INTRODUCTION

Hypertension is a major risk factor for cardiovascular disease. Globally, 54% of stroke and 47% of ischemic heart disease are attributable to high blood pressure.^1^ Overall, about 80% of the attributable burden for cardiovascular disease due to hypertension occurred in low-income and middle-income economies.^1^

Low socioeconomic status (SES) is associated with high blood pressure and its related cardiovascular disease morbidity and mortality.^2^^–^^4^ Some studies reported that hypertension was the primary reason for socioeconomic inequality in mortality. Therefore, policy efforts to reduce socioeconomic inequality in cardiovascular risk factors are needed.^5^^,^^6^ The social gap in mortality from hypertension-related disease has been attributed to differences in behavioral risk factors, demand for and access to services, treatment rates, and psychosocial factors.^7^

The impact of SES on hypertension might be due to an association between SES and blood pressure, to SES differences in hypertension care, or to SES differences in established risk factors for hypertension.^7^ In this study, we examined the association between SES and hypertension care such as treatment and control.

SES plays an important role among the factors that explain inequalities in hypertension awareness, treatment, and control.^2^ Socioeconomic status itself might be independently associated with hypertension care, as it affects factors such as disease knowledge, disease awareness, health-promoting behaviors, access to health care, and family and social support.

From a public health perspective, hypertension prevalence and care have different meanings: one relates to primary care and the other to secondary prevention. Thus, both are important, and we need to better understand how SES influences hypertension. Although SES can be considered an important factor in hypertension care, few studies have investigated socioeconomic disparities in hypertension care in Asian countries.^4^^,^^7^ Most data on socioeconomic inequalities in hypertension were collected in Western industrialized societies,^8^^–^^13^ and the relationship between SES and hypertension care varies across countries and study populations.^4^^,^^7^

South Korea is one of the world’s most rapidly aging societies. It is currently experiencing a transition to a westernized lifestyle, which has increased rates of cardiovascular disease. Cerebrovascular disease is the second most common cause of death, and heart disease is the third.^14^ Among Koreans, high blood pressure has a greater impact than other components of the metabolic syndrome on cardiovascular mortality.^15^ In addition, socioeconomic differentials were found in total mortality and cardiovascular mortality in Korea.^6^ Disparities in hypertension prevalence, awareness, treatment, and control could contribute to these differentials.

Although hypertension care has improved throughout the world,^9^ the association between SES and hypertension care is complex and unclear. Thus far, few studies have carefully examined this association in Korea or other Asian countries. We therefore investigated socioeconomic inequalities in hypertension prevalence, treatment, and control among middle-aged Koreans.

## METHODS

### Data sources and study subjects

The data were derived from the 2007 and 2008 Korean National Health and Nutrition Examination Survey (KNHANES) on Korean civilians, conducted by the Korean Centers for Disease Control and Prevention. KNHANES was a cross-sectional survey that used a stratified, multistage, probability sampling design to represent the entire Korean population. The sampling frame was based on the 2005 National Census Registry, and the survey used complex sampling on a rolling sample basis.^16^ The primary sampling unit was the administrative district, and there were 200 primary sampling units in each year. Three hundred sampling frames (100 in 2007, 200 in 2008) from the primary sampling units were randomly sampled, and a total of 18 983 individuals (6900 households) from these sampling frames were included in the survey. The response rate was 75.53%. We used the data from 4275 adults aged 40 to 64 years (1849 men, 2426 women). In consideration of the applicability of SES indicators, adults younger than 40 years and those older than 64 years were not included in the analysis, as they might not have been economically active. The study was conducted in accordance with the Declaration of Helsinki.

### Basic questionnaire and anthropometric measurement

The survey was divided into the health interview survey and health examination study. Nurses were specially trained to obtain blood pressure readings, collect serum, and obtain anthropometric measurements. Body weight and height were measured while subjects wore light clothing and no shoes or socks. Body mass index (BMI) was calculated by dividing weight in kilograms by height in meters squared, and categorized as underweight (<18.5), normal (18.5–27.5), or overweight (≥27.5). Self-reported smoking, alcohol consumption, and physical activity were estimated from questionnaire responses. Smoking status was categorized as current smoker, former smoker, and never smoker. Alcohol consumption was estimated from the frequency of high-risk alcohol consumption and classified as rarely or never, less than once per month, or at least once per month. On the basis of standards developed by the World Health Organization, high-risk consumption was defined as an alcohol consumption of 61 grams or more among men, or 41 grams or more among women, on a given day.^17^ This corresponds to the alcohol content in approximately 7 drinks of *soju* in men, and 5 drinks of *soju* in women (*soju* is one of the most popular alcoholic beverages in Korea and was used representatively in the health interview survey in Korea). Physical activity was classified as none, 1 to 2 times per week, or more than 2 times per week.

### SES indicators

Education, income, and occupation levels were used as indicators of SES. Data on these indicators were obtained from the Health Interview survey, which was done by the interviewer-assisted method. Education level was grouped into 3 categories: less than 7 years (elementary school graduates), 7 to 12 years (middle or high school graduates), or more than 12 years (college graduates). Income was based on household equivalent income, which was calculated by dividing average monthly household income by the square root of household size^18^ and was classified by using tertile distribution as low, middle, or high. Occupation was based on the Korean standard for classifying occupations—which is derived from the International Standard Classification of Occupations of the International Labor Organization^19^—and categorized as non-manual, manual, or others according to the definition of employment status used by the Korean National Statistical Office. Non-manual occupations included managers, professionals, and clerks, while manual occupations included services and sales workers, agricultural and fishery workers, craft and related trade workers, plant and machine operators and assemblers, and elementary occupations (ie, those with modest educational requirements). Those not in the labor market (unemployed, retired, students, and homemakers) were categorized as others.

### Blood pressure measurement

Blood pressure was measured with a mercury sphygmomanometer (Baumanometer; WA Baum Co Inc, New York, NY, USA) in the sitting position after a 10-minute rest period. The subjects were asked to refrain from smoking or caffeine for 30 minutes before the measurement. The first appearance of sound (the first Korotkoff sound) was used for systolic blood pressure (SBP), and the disappearance of sound (the fifth Korotkoff sound) was used for diastolic blood pressure (DBP). Three readings were taken at 30-second intervals, and the average of the latter 2 was used in the analysis. Hypertension was defined as 1 of the following: SBP of 140 mm Hg or higher and/or DBP of 90 mm Hg or higher, use of antihypertensive drugs, or an answer of “yes” to the survey question, “Have you had hypertension more than 3 months in the last 12 months?”. Untreated hypertension was defined as an answer of “No” to the question, “Are you now taking an antihypertensive drug?”, by a person with hypertension. Uncontrolled hypertension was defined as SBP of 140 mm Hg or higher or DBP of 90 mm Hg or higher. Both pharmaceutical and non-pharmaceutical measures were acceptable as methods to control hypertension.

### Statistical analysis

Data analysis was performed by using SAS (version 9.1; SAS Institute, Cary, NC, USA). All data were analyzed separately for men and women. The characteristics of study subjects are presented as mean (SD) or as numbers and percentages. The prevalence, treatment, and control rate of hypertension for each SES group were calculated by direct age standardization (5 year intervals), with the total study population as the standard. Odds ratios (ORs) and 95% CIs for prevalence, treatment, and control were estimated by multiple logistic regression adjusted for age alone and after adjustment for a number of other confounding variables, including marital status, residential area, obesity, smoking, alcohol consumption, and physical activity. The analysis reflects the complex survey sample design, which includes stratification, clustering, and unequal weighting.^20^ All statistical analyses were 2-tailed, and *P* values less than 0.05 were considered statistically significant.

## RESULTS

Tables 1 and 2 show the baseline characteristics of the study population by hypertension status and sex. There were significant differences in SES with regard to hypertension status in both sexes. Individuals with hypertension had lower SES than those without hypertension, and those with uncontrolled hypertension had lower SES than those with controlled hypertension. These differences in SES among groups were more obvious among women. Table 3 shows age-adjusted prevalences of untreated, uncontrolled, and all hypertension, classified by SES and sex. The prevalence of hypertension was calculated for the total study population, and the rates of untreated and uncontrolled were calculated among hypertensives. In both sexes, lower SES was associated with higher prevalences of hypertension and untreated and uncontrolled hypertension. Table 4 shows age-adjusted and multivariate ORs for hypertension, and for untreated and uncontrolled hypertension, by SES and sex. Multivariate analysis was adjusted for age, SES, marital status, residential area, and behavioral risk factors. Among men, the age-adjusted ORs for untreated and uncontrolled hypertension significantly increased as education level decreased. Among women, the age-adjusted ORs for prevalence of hypertension increased as education level decreased, and female manual workers had a higher OR for uncontrolled hypertension than did women in other occupations.

**Table 1.** Characteristics of male subjects, by hypertension status

	All men	No hypertension	Controlled hypertension	Uncontrolled hypertension	*P*-value^a^
	(*n* = 1849)	(*n* = 1258)	(*n* = 225)	(*n* = 366)	
Age (years)	51.2 ± 7.2	50.4 ± 7.1	55.2 ± 6.4	51.5 ± 6.9	
SBP (mm Hg)	119.9 ± 15.6	113.6 ± 10.9	121.1 ± 10.4	140.7 ± 13.7	
DBP (mm Hg)	79.8 ± 10.4	75.9 ± 7.7	78.5 ± 7.5	94 ± 7.1	
Education (years)					
>12	509 (27.6)	379 (30.2)	53 (23.7)	77 (21.1)	0.0022
7–12	969 (52.5)	648 (51.6)	116 (51.8)	205 (56.2)	
<7	367 (19.9)	229 (18.2)	55 (24.6)	83 (22.7)	
Income tertile					
High	635 (35.4)	440 (36.0)	74 (33.8)	121 (34.2)	0.0117
Middle	659 (36.7)	469 (38.4)	80 (36.5)	110 (31.1)	
Low	501 (27.9)	313 (25.6)	65 (29.7)	123 (34.8)	
Job					
Non-manual	462 (25.1)	332 (26.5)	50 (22.2)	80 (21.9)	0.0008
Manual	1106 (60.0)	756 (60.3)	122 (54.2)	228 (62.3)	
Others	276 (15.0)	165 (13.2)	53 (23.6)	58 (15.9)	
Marital status					
Married	1648 (89.76)	1126 (90.3)	199 (88.8)	323 (88.5)	0.5394
Others^b^	188 (10.2)	121 (9.7)	25 (11.2)	42 (11.5)	
Residential area					
Urban	1330 (71.2)	911 (72.4)	156 (69.3)	263 (71.9)	0.6377
Rural	519 (28.1)	347 (27.6)	69 (30.7)	103 (28.1)	
Body mass index (kg/m^2^)					
<18.5	37 (2.0)	29 (2.3)	3 (1.4)	5 (1.4)	<0.0001
18.5–27.5	1583 (86.2)	1110 (88.7)	183 (82.4)	290 (79.9)	
≥27.5	217 (11.8)	113 (9.0)	36 (16.2)	68 (18.7)	
Smoking					
Never	286 (15.5)	198 (15.8)	36 (16.0)	52 (14.2)	0.1100
Former	759 (41.1)	495 (39.4)	108 (48.0)	156 (42.6)	
Current	801 (43.3)	562 (44.8)	81 (36.0)	158 (43.2)	
Exercise (times/week)					
None	1092 (59.1)	753 (60.0)	133 (59.1)	206 (56.4)	0.4777
1–2	420 (22.7)	287 (22.9)	52 (23.1)	81 (22.2)	
>2	333 (18.0)	215 (17.1)	40 (17.8)	78 (21.4)	
Alcohol^c^					
Rarely or never	1043 (56.4)	207 (16.6)	47 (21.0)	37 (10.2)	<0.0001
<1/month	503 (27.2)	372 (29.8)	60 (26.8)	71 (19.5)	
≥1/month	291 (15.7)	670 (53.6)	117 (52.2)	256 (70.3)	

Abbreviations: DBP, diastolic blood pressure; SBP, systolic blood pressure.

Data are mean ± SD, or *n* (%).

^a^*P*-values indicate the difference among individuals with no hypertension, controlled hypertension, and uncontrolled hypertension.

^b^Including widowed and divorced persons.

^c^Frequency of high-risk alcohol consumption (≥61 grams on 1 occasion).

**Table 2.** Characteristics of female subjects, by hypertension status

	All women	No hypertension	Controlled hypertension	Uncontrolled hypertension	*P*-value^a^
	(*n* = 2426)	(*n* = 1800)	(*n* = 313)	(*n* = 313)	
Age (years)	51.0 ± 7.1	50.0 ± 6.9	55.7 ± 6.2	53.8 ± 6.6	
SBP (mm Hg)	115.8 ± 17.0	109.8 ± 11.5	120.6 ± 10.7	146.0 ± 14.1	
DBP (mm Hg)	75.2 ± 10.3	72.0 ± 7.9	76.9 ± 7.2	91.6 ± 9.0	
Education (years)					
>12	317 (13.1)	285 (15.9)	15 (4.8)	17 (5.5)	<0.0001
7–12	1243 (51.3)	969 (53.9)	140 (44.9)	134 (43.0)	
<7	863 (35.6)	545 (30.3)	157 (50.3)	161 (51.6)	
Income tertile					
High	733 (31.2)	600 (34.3)	66 (21.9)	67 (22.0)	<0.0001
Middle	791 (33.6)	597 (34.2)	90 (30.1)	104 (34.1)	
Low	829 (35.2)	550 (31.5)	145 (48.2)	134 (43.9)	
Job					
Non-manual	226 (9.3)	192 (10.7)	22 (7.0)	12 (3.8)	<0.0001
Manual	1084 (44.7)	807 (44.9)	117 (37.4)	160 (51.1)	
Others	1113 (45.9)	798 (44.4)	174 (55.6)	141 (45.1)	
Marital status					
Married	1997 (82.8)	1509 (84.3)	240 (77.2)	248 (79.7)	0.0030
Others^b^	416 (17.2)	282 (15.8)	71 (22.8)	63 (20.3)	
Residential area					
Urban	1792 (73.9)	1347 (74.8)	224 (71.6)	221 (70.6)	0.1779
Rural	634 (26.1)	453 (25.2)	89 (28.4)	92 (29.4)	
Body mass index (kg/m^2^)					
<18.5	54 (2.2)	49 (2.7)	1 (0.3)	4 (1.3)	<0.0001
18.5–27.5	2060 (85.3)	1582 (88.0)	236 (75.9)	242 (78.8)	
≥27.5	300 (12.4)	165 (9.2)	74 (23.8)	61 (19.9)	
Smoking					
Never	2244 (92.5)	1672 (92.9)	294 (93.9)	278 (89.1)	0.0262
Former	78 (3.2)	60 (3.3)	8 (2.6)	10 (3.2)	
Current	103 (4.3)	68 (3.8)	11 (3.5)	24 (7.7)	
Exercise (times/week)					
None	1705 (70.3)	1244 (69.1)	233 (74.4)	228 (73.6)	0.0908
1–2	366 (15.1)	293 (16.3)	36 (11.5)	37 (11.9)	
>2	351 (14.5)	262 (14.6)	44 (14.0)	45 (14.5)	
Alcohol^c^					
Rarely or never	922 (38.0)	654 (36.5)	139 (44.4)	129 (41.6)	0.0004
<1/month	1120 (46.2)	866 (48.4)	137 (43.8)	117 (37.7)	
≥1/month	372 (15.3)	271 (15.1)	37 (11.82)	64 (20.7)	

Abbreviations: DBP, diastolic blood pressure; SBP, systolic blood pressure.

Data are mean ± SD, or *n* (%).

^a^*P*-values indicate the difference among individuals with no hypertension, controlled hypertension, and uncontrolled hypertension.

^b^Including widowed and divorced persons.

^c^Frequency of high-risk consumption (≥41 grams on 1 occasion).

**Table 3.** Age-adjusted proportions^a^ of hypertension and untreated and uncontrolled hypertension, by socioeconomic status

	Men			Women		
		
	Hypertension	Untreated hypertension	Uncontrolled hypertension	Hypertension	Untreated hypertension	Uncontrolled hypertension
Education (years)						
>12	28.1 (28.0–28.2)	33.8 (33.7–34.0)	49.7 (49.5–49.9)	13.4 (13.3–13.5)	25.1 (24.8–25.4)	53.5 (52.8–54.2)
7–12	33.6 (33.5–33.6)	48.6 (48.4–48.7)	63.8 (63.7–63.9)	27.2 (27.1–27.3)	29.4 (29.3–29.5)	44.6 (44.4–44.7)
<7	37.0 (36.8–37.2)	55.7 (55.4–55.9)	67.7 (67.4–68.0)	30.8 (30.7–30.8)	42.0 (41.8–42.2)	56.2 (56.0–56.4)
Income tertile						
High	30.9 (30.8–31.0)	44.2 (44.0–44.3)	58.5 (58.4–58.7)	23.6 (23.5–23.7)	30.0 (29.9–30.2)	43.9 (43.7–44.1)
Middle	30.6 (30.5–30.6)	44.2 (44.0–44.3)	58.9 (58.7–59.0)	27.9 (27.8–27.9)	34.8 (34.7–35.0)	52.7 (52.5–52.9)
Low	37.8 (37.7–37.8)	51.4 (51.3–51.6)	67.2 (67.0–67.4)	29.9 (29.9–30.0)	36.7 (36.6–36.9)	51.3 (51.1–51.4)
Job						
Others	35.3 (35.2–35.4)	43.7 (43.4–43.9)	63.5 (63.2–63.7)	28.1 (28.1–28.2)	35.0 (34.9–35.1)	47.6 (47.5–47.7)
Manual	31.9 (31.9–32.0)	51.8 (51.7–51.9)	65.8 (65.7–66.0)	26.8 (26.7–26.9)	38.0 (37.9–38.2)	56.9 (56.7–57.0)
Non-manual	29.5 (29.4–29.6)	39.9 (39.8–40.1)	48.7 (48.5–48.9)	22.7 (22.5–22.9)	19.0 (18.8–19.3)	18.7 (18.4–18.9)

^a^Calculated by direct age standardization (5 year interval) using the total study population as the standard.

**Table 4.** Age-adjusted and multivariate odds ratios (95% CI)^a^ for hypertension, untreated hypertension, and uncontrolled hypertension by socioeconomic status

	Hypertension		Untreated hypertension		Uncontrolled hypertension	
		
	Age-adjusted	Multivariate	Age-adjusted	Multivariate	Age-adjusted	Multivariate
	Men					
	
Education (years)						
>12	1	1	1	1	1	1
7–12	1.29 (0.98–1.70)	1.30 (0.84–2.04)	2.19 (1.27–3.75)	2.14 (1.18–3.90)	1.94 (1.15–3.26)	1.37 (0.76–2.47)
<7	1.42 (0.98–2.07)	1.18 (0.88–1.58)	3.18 (1.65–6.15)	2.98 (1.42–6.28)	2.43 (1.26–4.67)	1.61 (0.78–3.32)
*P* for trend	0.1474	0.2439	0.0115	0.0041	0.3093	0.1971
Income tertile						
High	1	1	1	1	1	1
Middle	0.99 (0.76–1.30)	0.94 (0.71–1.25)	1.04 (0.63–1.71)	0.87 (0.52–1.46)	1.15 (0.71–1.88)	0.95 (0.57–1.58)
Low	1.38 (1.01–1.87)	1.26 (0.88–1.81)	1.33 (0.78–2.28)	1.19 (0.63–2.24)	1.55 (0.91–2.67)	1.34 (0.73–2.45)
*P* for trend	0.2303	0.2049	0.4581	0.5958	0.2552	0.3640
Job						
Others	1	1	1	1	1	1
Manual	0.84 (0.62–1.15)	0.85 (0.58–1.26)	1.60 (0.96–2.65)	1.38 (0.76–2.53)	1.15 (0.69–1.91)	0.97 (0.54–1.73)
Non-manual	0.80 (0.55–1.17)	0.97 (0.60–1.58)	1.02 (0.58–1.81)	1.25 (0.62–2.50)	0.61 (0.33–1.10)	0.64 (0.33–1.27)

	Women					
	
Education (years)						
>12	1	1	1	1	1	1
7–12	2.13 (1.37–3.29)	1.75 (1.10–2.78)	0.67 (0.29–1.55)	0.53 (0.22–1.28)	0.87 (0.39–1.94)	0.84 (0.36–1.96)
<7	2.64 (1.67–4.18)	1.88 (1.12–3.16)	1.30 (0.56–3.05)	1.16 (0.45–2.96)	1.47 (0.64–3.37)	1.46 (0.59–3.63)
*P* for trend	0.0323	0.0173	0.5289	0.7645	0.4319	0.4145
Income tertile						
High	1	1	1	1	1	1
Middle	1.41 (1.05–1.90)	1.28 (0.93–1.76)	1.09 (0.63–1.87)	1.07 (0.60–1.90)	1.43 (0.88–2.33)	1.34 (0.82–2.20)
Low	1.46 (1.06–2.01)	1.16 (0.81–1.64)	1.26 (0.77–2.05)	1.08 (0.62–1.87)	1.33 (0.82–2.16)	1.12 (0.67–1.88)
*P* for trend	0.5373	0.4187	0.4077	0.7857	0.5737	0.6552
Job						
Others	1	1	1	1	1	1
Manual	0.98 (0.79–1.22)	0.89 (0.71–1.13)	1.09 (0.72–1.64)	1.09 (0.69–1.72)	1.46 (1.01–2.10)	1.50 (1.02–2.22)
Non-manual	0.60 (0.38–0.94)	0.65 (0.41–1.05)	0.57 (0.26–1.25)	0.87 (0.36–2.14)	0.35 (0.16–0.78)	0.51 (0.22–1.19)

^a^Adjusted for age, marital status, residential area, obesity, smoking, alcohol consumption, and physical activity.

Regarding multivariate ORs, among men, the ORs for untreated hypertension significantly increased as education level decreased, but the ORs for any hypertension and uncontrolled hypertension did not significantly differ by education level. Among women, low education was linked to a higher OR for any hypertension, but the ORs for untreated and uncontrolled hypertension did not significantly differ by education level. Among women, manual workers had a higher OR for uncontrolled hypertension as compared with those in other occupations. Income level was not significantly associated with hypertension treatment or control in either sex.

## DISCUSSION

Although SES is thought to be an important factor in hypertension care, few studies have investigated socioeconomic disparities in hypertension care in Asian countries. In this study, we observed socioeconomic disparities in hypertension prevalence and care among middle-aged Koreans. Among the SES indicators, education level was the most important explanatory variable of inequalities in hypertension prevalence and care. Although we observed an educational disparity in hypertension prevalence among women, the educational disparity in treatment was more obvious among men.

Our data on hypertension prevalence were consistent with the findings of previous studies, which found that, as SES declined, hypertension was more prevalent among women than among men.^4^^,^^21^^–^^25^ The reason for this discrepancy between sexes might be the greater concurrent risks of metabolic disease and psychosocial stress (such as hostility, depression, and social isolation) for women of low SES as compared with men of low SES.^4^^,^^26^^–^^28^ Although hypertension has both biological and lifestyle risk factors, its treatment and control depend on adherence, which encompasses acceptance, persistence and compliance.^29^ Putative barriers to adherence are low SES, poor geographic accessibility, characteristics of the health insurance system, poor doctor–patient communication, cost of antihypertensive therapy, and adverse effects of drugs.^4^^,^^30^

The association between hypertension care and SES is complex, and the results of this study differ somewhat from those of previous studies.^8^^,^^10^^,^^11^^,^^26^^,^^30^^–^^34^ In some earlier studies, lower hypertension awareness, treatment, and control were observed among patients with low SES.^32^^,^^33^ Other studies observed socioeconomic disparities in hypertension prevalence and awareness but not in treatment or control of diagnosed hypertension.^11^ In the United States, lack of insurance was associated with poor hypertension control among treated hypertensive patients, which was likely related to differences in appropriate treatment adherence.^35^ In a French study, therapeutic adherence was lower among individuals in lower educational level and low occupational categories, but treatment rate was unrelated to those variables.^10^ In China, people in rural areas tend to have a low education level and limited health education; thus, poor knowledge of hypertension was significantly associated with poor hypertension control.^30^^,^^36^ In a multiethnic Asian population, higher education level was associated with reduced awareness, and individuals with low SES were more likely to be treated but had poorer control.^31^ A possible reason for the reduced awareness and treatment among individuals with high SES is that the affluent segment of the population is preoccupied with career development.^31^

We used education, income, and occupation levels as indicators of different dimensions of SES. There were statistically significant associations between education and hypertension treatment in men and between education and prevalence in women. In addition, occupation and hypertension control were associated in women. However, we did not find that people with higher incomes were more likely to have treated or controlled hypertension. A possible explanation for these findings is that hypertension care is related to adherence rather than to material resources alone. Under government-mandated health coverage, beginning at age 40 years, Koreans undergo health screening every 2 years as part of the national health screening program. Therefore, economic resources might not be the main determinant of hypertension care in the Korean system. However, education level might strongly influence adherence through not only economic support but also through knowledge of hypertension, health behavior, and levels of social and psychosocial support. Education might also have been a more robust indicator than other SES indicators because of inaccuracy and ambiguity in determining individual incomes and occupational status, which could have contributed to the weak associations of those variables with hypertension prevalence and treatment. Income can fluctuate over time and is thus susceptible to misclassification. Furthermore, household income might not accurately reflect individual consumption. Therefore, disposable income could be the real determining factor in private consumption, or wealth may be a proxy for permanent income that influences the consumption or health behavior of individuals.

Regarding associations between occupation and hypertension care, we found that women with manual jobs had a higher OR for uncontrolled hypertension than did those with other jobs. However, we cannot conclude that female manual workers are more susceptible than male manual workers to uncontrolled hypertension. In fact, the proportion of uncontrolled hypertension was 65.8% among male manual workers and 56.9% among female manual workers. In previous studies, the relationship between occupational stress and hypertension or coronary heart disease varied by race and ethnicity.^37^^–^^44^ In the United States, job strain was not associated with poor blood pressure control.^38^ Similarly, in the United Kingdom, hypertension did not explain the link between job strain and coronary heart disease.^42^ Among Asian countries, China has been as quick to industrialize as South Korea, and job stress in China was associated with SBP among working women. In Japan, the level of job strain was correlated with hypertension prevalence among men but not among women.^39^^,^^40^^,^^43^ Poor hypertension control among manual worker may be due to the presence of more barriers to blood pressure control. Workers engaged in low-paid, unskilled manual labor and service-sector jobs are struggling with psychosocial stress from financial strain, job insecurity, and low perceived control at work.^45^ These factors might affect therapeutic adherence and hypertension control.^34^

The present study has certain limitations that must be considered. First, our study used interviewer-assisted survey data to assess SES and a self-reported questionnaire to assess behavioral risk factors. Because these methods could lead to underestimation or overestimation of outcomes, it is imperative that these data are assessed for validity and reliability. Nevertheless, this was a large-scale national survey that represented the entire Korean population. In addition, a previous study evaluated the reliability of the educational and occupational categories used in this survey.^46^ Regarding income level, it would be better to collect objective individual income information, such as from the database for Korean National income tax reports. Regarding alcohol consumption, our categorization was based on the frequency of high-risk alcohol consumption, which could be inadequate for assessing alcohol-related harm. However, our analysis was limited to the fixed, stored data derived from the Korean National Health Survey. Although the questionnaire was carefully designed, we cannot exclude the possibility of inaccurate self-reporting. Second, the survey sampling frame includes only the non-institutionalized population, which could have led to underestimation of the socioeconomic disparities of hypertension care, especially among individuals in nursing homes, hospitals, and other institutions who might have more severe hypertension. However, it is unlikely that this problem biased the results to a significant degree, because individuals older than 65 years, who constitute the majority of institutional residents, were not included in the analysis. Third, as in most epidemiologic studies, the present cross-sectional study design does not permit causal inferences. Residual and unmeasured covariates could also affect evaluation of outcomes. Although we tried to minimize the effects of those variables, data on family history, severity of hypertension, and types of antihypertensive drugs were not available. We believe that the independent effect of SES on hypertension prevalence and care, after adjusting for covariates, is due to knowledge, attention, health information-seeking behavior, attitude, access to medical institute, and familial and social support for prevention and management of hypertension.

In conclusion, this study showed that SES was an important factor in high blood pressure and that education was the most robust socioeconomic indicator of hypertension prevalence and care. Sex-based socioeconomic disparities in hypertension prevalence and care remain significant among middle-aged Koreans, despite recent advances in medical treatment. Hence, comprehensive health policies that effectively reduce socioeconomic disparities in hypertension care should be developed and promoted.
